# Mendelian randomization analyses of associations between breast cancer and bone mineral density

**DOI:** 10.1038/s41598-023-28899-0

**Published:** 2023-01-31

**Authors:** Hong Wu, Hui Wang, Di Liu, Zhibing Liu, Weiming Zhang

**Affiliations:** 1 Department of Oncology, Wuming Hospital of Guangxi Medical University, Nanning, 530021 Guangxi China; 2 Department of Research, Guangxi Medical University Cancer Hospital, Nanning, Guangxi China; 3 The First Affiliated Hospital of Guangxi Medical University, Nanning, 530021 Guangxi China

**Keywords:** Cancer, Cancer genomics

## Abstract

The purpose of this study was to verify whether there is a causal relationship between breast cancer and bone mineral density (BMD). Summary statistics for exposures and outcomes were obtained from corresponding genome-wide association studies. The bidirectional and multivariate mediated Mendelian randomization (MR) analyses were performed. In the bidirectional MR analysis, breast cancer might reduce the BMD of the heel (HE-BMD) (FDR = 1.51 × 10^−4^) as might its ER+ subtype (FDR = 1.51 × 10^−4^). From BMD to breast cancer, no significant association was found (FDR > 0.05). The mediating MR analysis showed that Higher free testosterone (FT) only mediated the causal relationship between breast cancer and HE-BMD by 2.9%; both ER+ type and FT were independent factors of HE-BMD (ER+: P = 0.021; FT: P = 6.88 × 10^−6^). Higher FT could increase the risk of breast cancer (FDR = 1.21 × 10^−3^) as could total testosterone (TT) (FDR = 5.81 × 10^−3^). Similarly, higher FT could increase the risk of ER+ subtype (FDR = 2.51 × 10^−6^) as could TT (FDR = 5.55 × 10^−4^). These results indicate that BMD is not a risk factor for breast cancer but breast cancer and its ER+ subtype are risk factors for BMD loss. Furthermore, higher FT and TT levels are associated with both an increased incidence of breast cancer and increased bone density.

## Introduction

Breast cancer is one of the most common malignant tumors that harm women's health, and its incidence increases gradually with age. Nearly half of newly diagnosed breast cancer cases are in patients aged 65 or older^1,2^. Osteoporosis is the most common in postmenopausal women and is characterized by low bone density, which increases the risk of fractures^3^. So the risk of breast cancer and osteoporosis increases as women get older. A recent study compared the bone mineral density (BMD) of women newly diagnosed with breast cancer with that of women without breast cancer. It demonstrated that women with newly diagnosed breast cancer have higher BMD than women with similar characteristics but without breast cancer, suggesting that higher BMD may be a risk factor for breast cancer^4^. Similarly, a study of women in South Korea and a study of women in southern Israel both found that women who have higher rates of breast cancer also have higher bone density^5,6^. However, these studies are observational, and the causal relationship between breast cancer and BMD has not been elucidated—do women with breast cancer have higher bone density? Or does higher bone density increase the risk of breast cancer? Other relevant research results are different; they show that higher or lower bone density is not associated with breast cancer and that BMD cannot predict the risk of breast cancer^7–9^.

There are many risk factors associated with breast cancer and BMD. Hormone levels may play an important role in breast cancer and osteoporosis^10,11^. Higher levels of testosterone and oestradiol have been associated with an increased risk of breast cancer in both pre- and postmenopausal women as well as women of different ethnic groups and have been more commonly reported in postmenopausal populations^12–16^. Higher testosterone and oestradiol levels can increase bone density and reduce the risk of osteoporosis^17–19^. Are hormone levels a potential link between osteoporosis and breast cancer? Since the causal relationship between osteoporosis and breast cancer is still unclear, and to explore the role of hormone levels, bidirectional, multivariate mediated Mendelian randomization (MR) methods were used in this study. Testosterone is an important female hormone that acts as both an essential precursor for estradiol biosynthesis and an androgen. Therefore, this study chose testosterone as the research object of hormone level. MR, an epidemiological causal inference method, is used to evaluate the potential causal influences of risk factors on outcomes by using genetic instrumental variables (IVs) and can reduce the bias caused by confounders^20^. Bidirectional MR can assess whether there is reverse causality between exposure and outcome, that is, whether outcome causes exposure. Multivariate mediated MR are used to investigate whether there is a mutual mediating effect between multiple factors on the same outcome. Heterogeneity, pleiotropy, and horizontal pleiotropy were used to assess the sensitivity of the results. There may be heterogeneity in the instrumental variables of different analysis platforms, experiments and populations. If instrumental variables do not directly affect the results through exposure factors, there will be pleiotropy. These may affect the robustness of MR results. The purpose of this study was to use MR methods to explore whether there is a causal relationship between breast cancer and BMD and whether hormone levels mediate the relationship between the two.

## Methods

### Genome-wide association study (GWAS) statistics of breast cancer

The instrumental variables (IVs) for breast cancer were extracted from the Breast Cancer Association Consortium (BCAC), which was a large-scale meta-analysis conducted using iCOGS, OncoArray and nine of the GWAS datasets^21^. The study reported genome-wide association results from 122,977 breast cancer patients of European ancestry and 105,974 controls as well as 14,068 breast cancer patients of East Asian ancestry and 13,104 controls. Only the subjects with European ancestry were included in our MR study. Analysis statistics from OncoArray and iCOGS were adjusted for country and study. For the OncoArray analysis, statistics were adjusted for country and 10 principal components. In our MR analysis, we included single nucleotide polymorphisms (SNPs) associated with breast cancer and its subtypes, oestrogen receptor-positive/negative (ER+/ER−), as IVs of exposure in European women, and they were also used as outcomes in the bidirectional MR analysis.

### GWAS statistics of BMD

BMD is an important basis for the clinical diagnosis of osteoporosis. In this study, BMD was used as the outcome of MR analysis and as exposure in bidirectional MR. The GWAS summary statistics related to BMD were selected from two large meta-analyses and assessed at four different bone sites. One GWAS included 53,236 individuals of European ancestry, including lumbar spine BMD (LS-BMD), femoral neck BMD (FN-BMD), and forearm BMD (FA-BMD), and the covariates were sex, age and weight^22^. The other GWAS included 426,824 European individuals for heel BMD (HE-BMD), and the covariates were sex, age and genotype^23^.

### GWAS statistics of female hormone levels

The GWAS summary statistics of hormone levels were obtained from a recent GWAS, which was a large-scale meta-analysis conducted using the UK Biobank^24^. The participants were 425,097 Europeans with sex-hormone binding globulin (SHBG), total testosterone (TT), and oestradiol statistics and 382,988 Europeans with free testosterone (FT) statistics. The researchers adjusted for age and BMI and disaggregated by sex. Since most of the women in the study were postmenopausal and their oestrogen levels were undetectable, the analysis was limited by a bias towards detecting age at menopause-associated loci. Therefore, oestradiol level assessment in women was not considered. In our MR study, the FT and TT of women were included as the study objects of hormone levels.

### Mendelian randomization design

Several observational studies have found an association between breast cancer and bone mineral density risk. Three reasonable hypotheses can be made about this association: (1) breast cancer is a risk factor for BMD change; (2) BMD is a risk factor for breast cancer; and (3) whether hormone levels play a mediating role between breast cancer and BMD.

**Figure 1:**
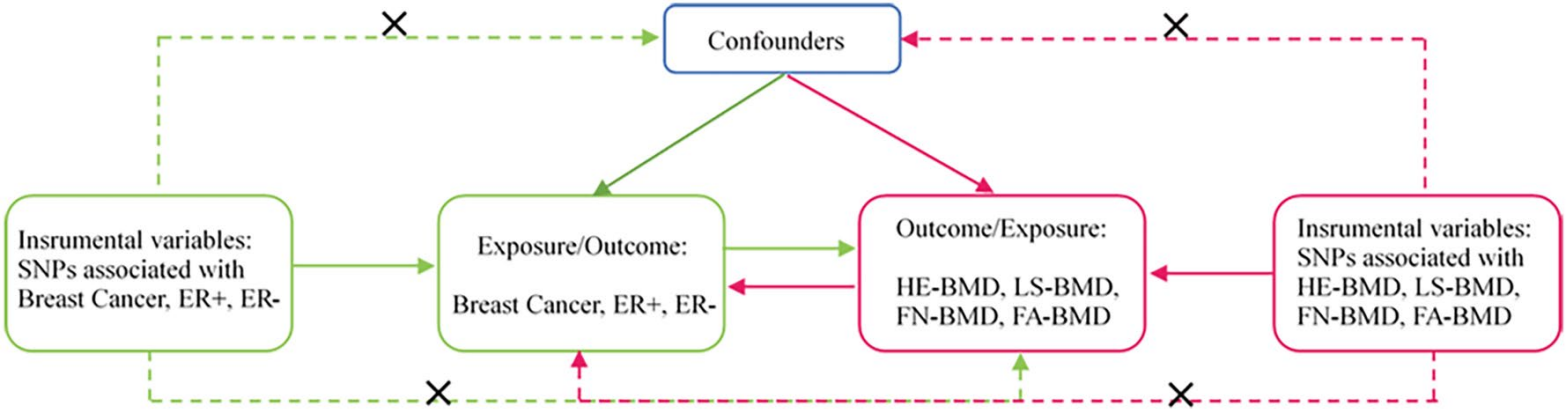
The framework of two sample bidirectional Mendelian randomization analysis. Notes: Mendelian randomization approach builds upon three important assumptions. 1: The genetic variations are strongly associated with exposure; 2: The genetic variations are not associated with either known or unknown confounders; 3: SNPs should influence risk of the outcome through the exposure, not through other pathways. The green line represented the Mendelian randomization analysis of the association of breast cancer and its subtypes with bone mineral density at different sites. The red line represented the Mendelian randomization analysis of the association of bone mineral density at different sites with breast cancer and its subtypes.

A bidirectional MR was used to test the first two hypotheses. For the first hypothesis, we first selected significantly correlated SNPs from GWAS results of breast cancer and ensured that these SNPs were independent of confounders and BMD. BMD was used as the outcome, and relevant SNP information was extracted from the corresponding GWAS data. BMD was used as exposure in the second hypothesis. SNPs significantly related to BMD were identified from GWAS to ensure that these SNPs were independent of confounders and were not directly related to breast cancer. Breast cancer was taken as the outcome. Bidirectional MR analysis was performed to clarify the causal relationship between breast cancer and BMD. A bidirectional MR analysis flow chart is shown in Fig. 1.

For the third hypothesis, the multivariate mediated MR analysis was performed to determine whether hormone levels played a role in the relationship between BMD and breast cancer^25^. For the proportion of intermediary factors, refer to the research method of Xu et al., first calculate the indirect effect, and then divide by the total effect^26^. The effect of exposure on mediating factors was determined as β1; After correcting for the effect of exposure, the effect of mediators on the results was β2; After correcting the mediating effect, the effect of exposure on the results was β3. β1 × β2 was the indirect effect and β3 + β1 × β2 was the total effect^26^.

### Mendelian randomization statistical analysis

SNPs significantly associated with exposure that achieved genome-wide significance (p value < 5 × 10^−8^) and had a minor allele frequency > 0.01 were identified in GWAS databases and used as IVs. The condition r^2^ = 0.01 and KB = 5000 was used to remove SNPs with linkage disequilibrium (LD). The phenotypic variation explained by SNPs was calculated as follows: R^2^ = 2 × beta^2^ × (1 − EAF) × EAF/SD^2^, with EAF = effect allele frequency and beta = the effect of each SNP on the exposures^27^. The F statistic (F = beta^2^/se^2^) was used to test the strength of the association between these SNPs and the exposure factors. SNPs with strong statistical power (F statistics > 10) were included. These screened SNPs were extracted from the outcome-related GWAS databases. Then, the effect value directions of the exposure data and the outcome data were unified, and the SNPs that were palindromic with the intermediate allele frequency > 0.45 and < 0.55 were removed^28^. And SNPs from the exposure data were discarded when they could not be found in the outcome data.

The inverse-variance weighted (IVW), weighted-median^29^, and MR-Egger^30^ were the main estimation methods for the MR statistical analysis to examine the causal relationship between the exposure and outcome. In addition, the MR-PRESSO^31^ method was used to detect outliers. Cochrane’s Q value was used to assess the heterogeneity^32^, and the MR-Egger intercept was used to detect horizontal pleiotropy^30^. When there was no heterogeneity or horizontal pleiotropy, the IVW method was used as the main effect size. When there was heterogeneity and no horizontal pleiotropy, the weighted-median method was dominant. When both heterogeneity and horizontal pleiotropy were present, MR-Egger was adopted. The false discovery rate (FDR) based on the Benjamini and Hochberg method was used to adjust the P values for multiple testing. The mRnd (https://cnsgenomics.shinyapps.io/mRnd/) was adopted to calculate the statistical power of MR.

All Mendelian randomization analyses were performed in R software version 4.1.1 using the “TwoSampleMR”^28^, “MR-PRESSO”^31^, and “MendelianRandomization”^33^ packages.

### Ethics approval and consent to participate

Ethical approval was not required because this study used the data from publicly available databases.

## Results

The number of SNPs selected as instrumental variables that were significantly correlated with exposure ranged from 4 to 1079. Their explained variances varied from 2.5 to 31.7%. The F statistics for each SNP and the general F statistics were all greater than 10 (Table 1).

**Table 1:** Summary statistics of exposure.

Exposure	NSNP	Sample	R^2^ (%)	F	People	PMID
Breast cancer	184	228,951	29.3	515.2	European, females	29059683
ER+	135	175,475	31.7	602.8	European, females	29059683
ER−	40	127,442	13.9	514.2	European, females	29059683
HE-BMD	1079	426,824	20.5	61.1	European, females, males	30598549
LS-BMD	25	28,498	4.6	54.9	European, females, males	26367794
FN-BMD	21	32,735	3.1	49.8	European, females, males	26367794
FA-BMD	4	8143	2.5	52.2	European, females, males	26367794
FT	150	180,386	5.7	72.1	European, females	32042192
TT	135	199,569	4.9	76.1	European, females	32042192

### Bidirectional MR analysis

**Figure 2:**
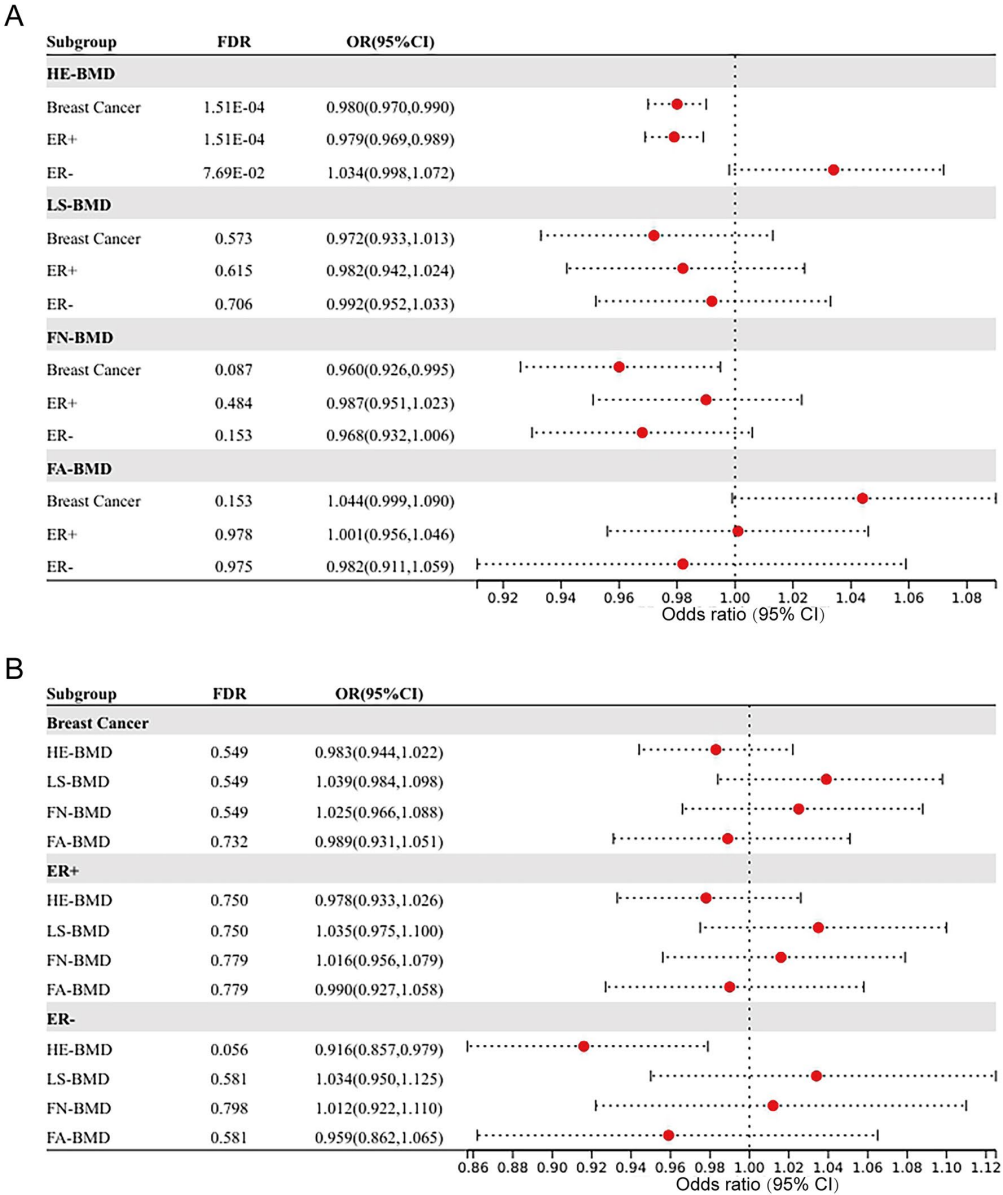
The forest plot of bidirectional Mendelian randomization results. (**A**) is the Mendelian randomization results of the association of breast cancer and its subtypes with bone mineral density at different sites. (**B**) is the Mendelian randomization results of the association of bone mineral density at different sites with breast cancer and its subtypes.

In the MR analysis from breast cancer to BMD, there was an inverse causal relationship between breast cancer, its ER+ subtype and HE-BMD. Breast cancer might reduce HE-BMD and was recognized as a risk factor for osteoporosis (OR 0.980, 95% CI 0.970–0.990, FDR = 1.51 × 10^−4^) as was the ER+ subtype (OR 0.979, 95% CI 0.969–0.989, FDR = 1.51 × 10^−4^) (Fig. 2A, Supplemental Table S1). The ER− subtype had no causal relationship with HE-BMD (OR 1.034, 95% CI 0.998–1.072, FDR = 7.69 × 10^−2^). No causal relationship was found between breast cancer, its subtype and BMD in other sites—LS, FN, FA. Heterogeneity was found in Mendelian randomization of breast cancer, ER+ subtype to HE-BMD, LS-BMD and FN-BMD. When heterogeneity existed in sensitivity analysis, the statistics of weighted median method were in the same direction as that of IVW models, and the weighted median method was selected as the main statistical effect. Horizontal pleiotropy was found for the ER− subtype to HE-BMD. The MR-Egger result was adopted as the main effect size. The original results of IVW, weighted-median, and MR-Egger between breast cancer and BMD can be found in Supplemental Table S2, together with the heterogeneity and pleiotropy tests.

In the MR analysis from BMD to breast cancer, no significant association was found (FDR > 0.05, Fig. 2B). There was heterogeneity in the MR statistics analysis of HE-BMD in breast cancer and its ER+ and ER− subtypes. No horizontal pleiotropy was found for BMD in any region to breast cancer in MR analysis. Supplemental Table S3 shows the results of IVW, weighted-median, and MR-Egger between BMD and breast cancer, together with the heterogeneity and pleiotropy tests.

### Multivariate mediated MR analysis

**Figure 3:**
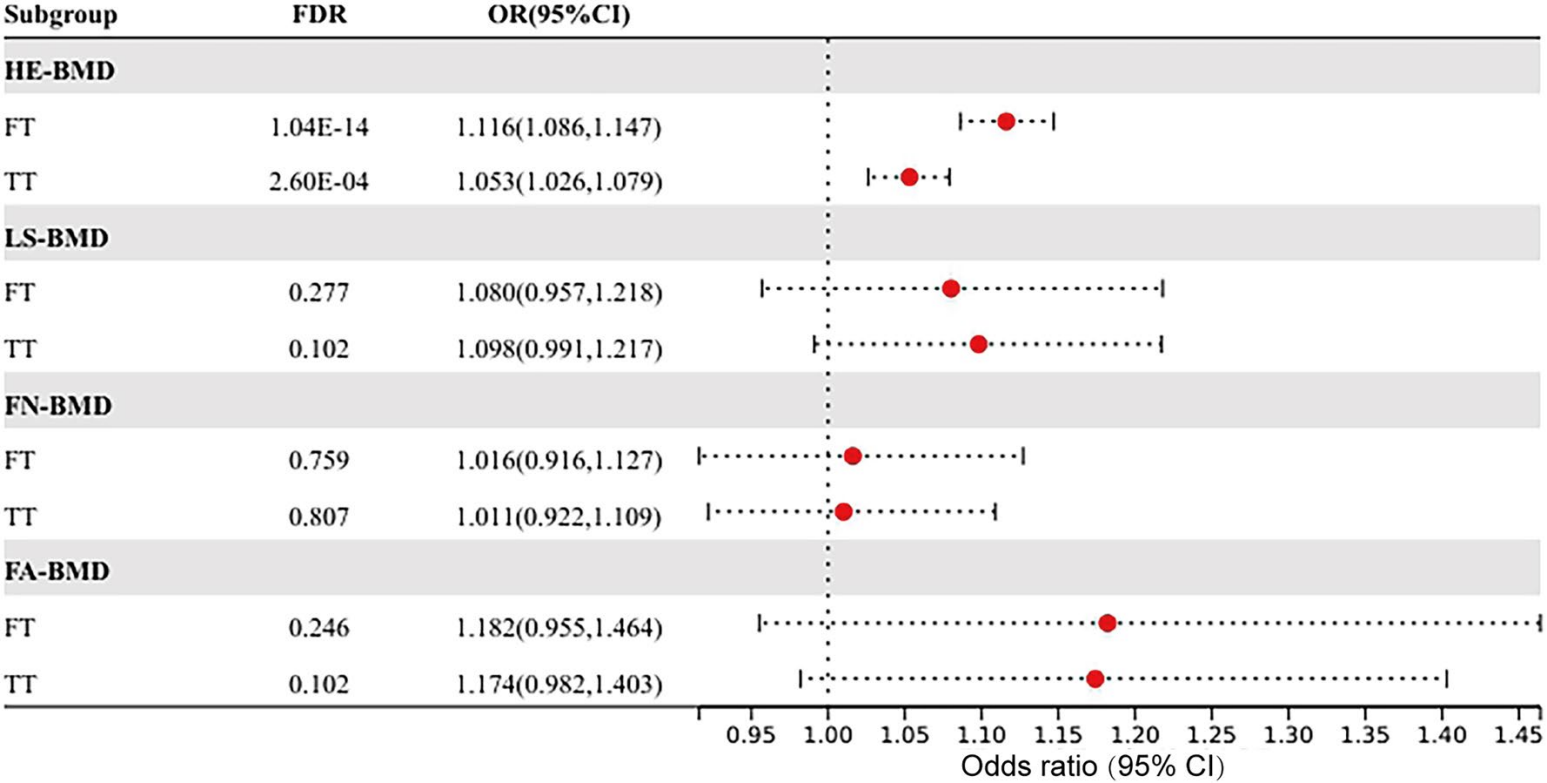
The forest plot of Mendelian randomization results between testosterone levels and bone mineral density at different sites.

**Figure 4:**
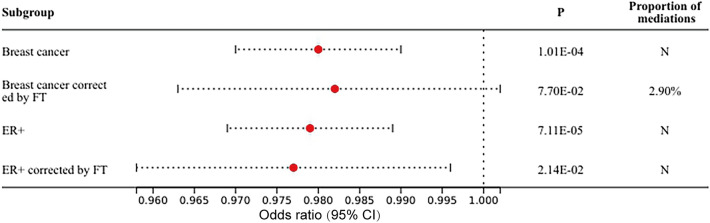
The forest plot and mediating ratios of breast cancer and its ER+ subtypes before and after FT adjustment on the bone density of the heel.

In the MR analysis of hormone levels to BMD, there was a positive causal relationship between FT, TT and HE-BMD. Higher FT could increase HE-BMD and was considered a protective factor for osteoporosis (OR 1.116, 95% CI 1.086–1.147, FDR = 1.04 × 10^−14^); similar results could be seen with TT (OR 1.039, 95% CI 1.013–1.066, FDR = 2.60 × 10^−4^) (Fig. 3, Supplemental Table S1). FT and TT had no causal relationship with LS-BMD, FN-BMD, or FA-BMD. Heterogeneity was found in the MR analysis of FT, TT to HE-BMD, LS-BMD, FN-BMD, and FA-BMD. The weighted median was adopted as the main method. No horizontal pleiotropy was found for any of the hormone levels to BMD in MR analysis. The original results of IVW, weighted-median, and MR-Egger between hormone levels and BMD can be found in Supplemental Table S4, together with the heterogeneity and pleiotropy tests. The multivariate MR analysis suggested that elevated FT may be an independent protective factor for HE-BMD (adjusted OR = 1.076, P = 0.033), while TT was not significant in multivariate MR model (adjusted OR = 1.023, P = 0.520). The results of mediating MR analysis suggested that FT mediated 71.5% of the causal relationship between TT and HE-BMD. There were causal relationships between breast cancer, its ER+ subtype, FT and HE-BMD. The multivariate MR analysis showed that breast cancer corrected for FT mediating factor had no significant adverse effect on HE-BMD (adjusted OR = 0.982, P = 0.077), while both its ER+ type and FT were independent factors of HE-BMD (ER+: adjusted OR = 0.977, P = 0.021; FT: adjusted OR = 1.111, P = 6.88 × 10^−6^). The mediating MR analysis showed that FT only mediated the causal relationship between breast cancer and HE-BMD by 2.9%. The effect of breast cancer and its ER+ subtypes adjusted FT on HE-BMD was shown in Fig. 4.

**Figure 5:**
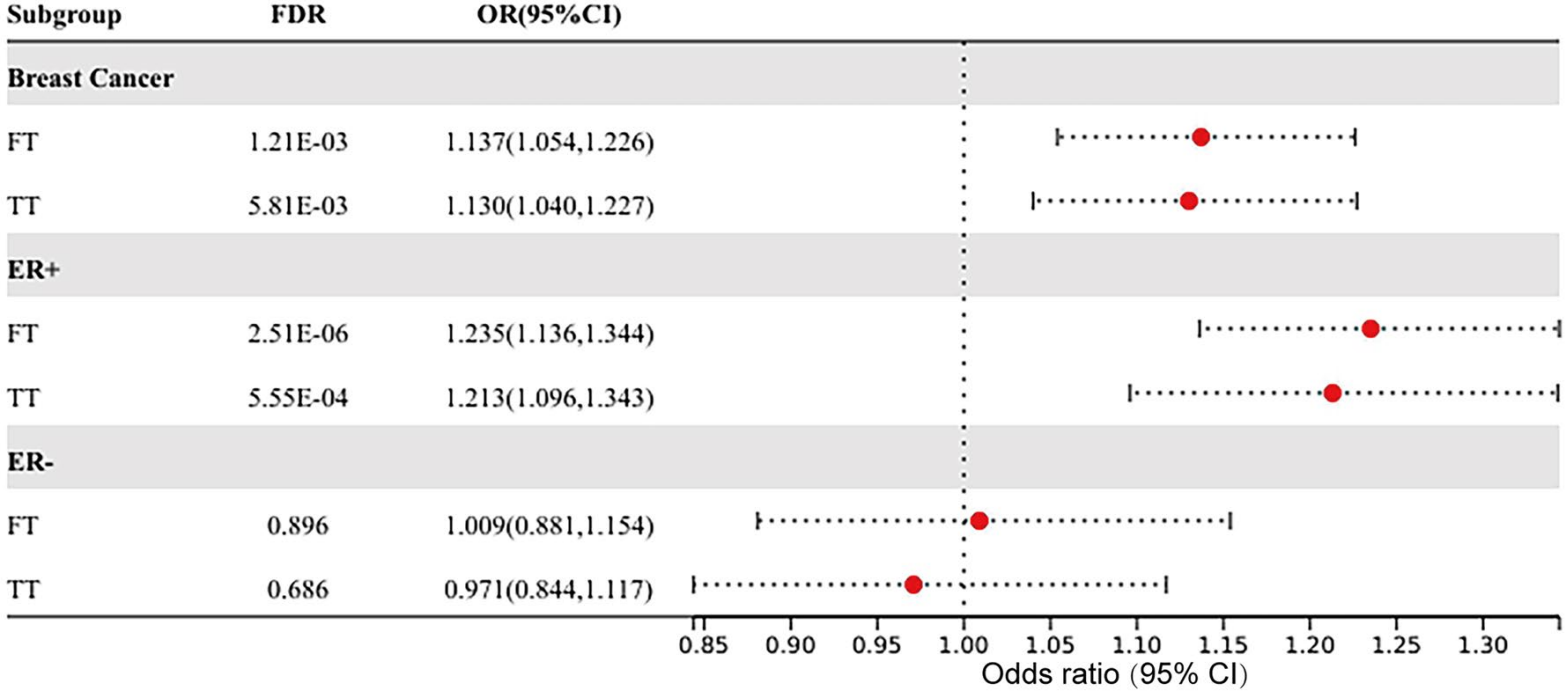
The forest plot of Mendelian randomization results between testosterone levels and breast cancer.

In the MR analysis of hormone levels in breast cancer, there was a positive causal relationship between FT and TT and breast cancer and its ER+ subtype. Higher FT could increase the risk of breast cancer (OR 1.137, 95% CI 1.054–1.226, FDR = 1.21 × 10^−3^) as could TT (OR 1.130, 95% CI 1.040–1.227, FDR = 5.81 × 10^−3^) (Fig. 5, Supplemental Table S1). Similarly, higher FT could increase the risk of ER+ subtype (OR 1.235, 95% CI 1.136–1.344, FDR = 2.51 × 10^−6^) as could TT (OR 1.213, 95% CI 1.096–1.343, FDR = 5.55 × 10^−4^) (Fig. 5, Supplemental Table S1). FT and TT had no causal relationship with the ER− subtype of breast cancer (FDR > 0.05, Fig. 5). Heterogeneity was found in the MR analysis of FT and TT in breast cancer and its ER+/ER− subtype, so the weighted-median results were adopted as the main effect. No horizontal pleiotropy was found for any of the hormone levels in breast cancer in MR analysis. The original results of IVW, weighted-median, and MR-Egger between hormone levels and breast cancer can be found in Supplemental Table S5, together with the heterogeneity and pleiotropy tests.

The statistical power for exposure to FT and TT to outcomes HE-BMD, breast cancer, and ER+ were all 100%; however, the statistical power of exposure to breast cancer and ER+ in HE-BMD outcomes was 74% and 69%, respectively.

## Discussion

Our MR study explored the causal relationships between breast cancer and BMD. In bidirectional MR, breast cancer and its ER+/ER− subtypes were used as exposure, and a negative causal relationship between breast cancer, ER+ subtype and HE-BMD was found. The incidence of breast cancer and its ER+ subtype may lead to a decrease in bone mineral density, which is consistent with many observational studies, although the statistical power of this causal relationship is insufficient. Several observational studies have explored changes in bone mineral density in breast cancer survivors. During 3 years of follow-up, women diagnosed with breast cancer lost up to 6.8% of their BMD. Chemotherapy and premenopausal status may be important risk factors for bone loss^34^. BMD in the hip and lumbar spine continues to decline after initiation of aromatase inhibitor therapy in breast cancer patients, and annual bone loss levels are elevated in women younger than 55 years of age^35^. A prospective cohort study found that young breast cancer survivors have an increased risk of developing osteoporosis compared with women without cancer^36^. Osteoporosis occurs during treatment and extends beyond breast cancer treatment, which is a long-term effect, but it can also occur after the end of breast cancer treatment as a late effect^37^. Osteopenia and osteoporosis in breast cancer survivors have been widely reported in clinical observational studies. Adjuvant chemotherapy and hormone therapy may be important causes of bone loss in breast cancer patients. Incidentally, hormone therapy is the main treatment for the ER+ subtype of breast cancer. In this MR analysis, breast cancer and ER+ subtypes were risk factors for decreased bone mineral density.

In several observational studies, women newly diagnosed with breast cancer, prior to any antitumor therapy, have been found to have higher BMD than women without breast cancer, and BMD is positively correlated with the incidence of breast cancer. High BMD is considered a biomarker of breast cancer risk^38–40^. However, bone mineral density was not found to be a risk factor for breast cancer in ourr MR Study. The same results are consistent with a recently published Mendelian Randomized study of bone density and breast cancer risk. The results of this study also provide no evidence to support a causal association between BMD and breast cancer, while considering that the association found in observational studies could be explained by polymorphic genetic variants that contribute to the pathology of osteoporosis and breast cancer^41^. Elevated hormone levels, including levels of oestradiol and testosterone, have been found to be associated with BMD and breast cancer in several studies^15,42^. So we further explored whether testosterone level mediate the relationship between breast cancer and BMD. Multivariate mediated MR studies had found that the effect of TT on HE-BMD was largely mediated by FT. The mediating effect of FT on the causal effect of breast cancer on HE-BMD was only 2.9%, which was equivalent to almost no mediating effect. There was no mediating effect of FT in the causal effect of ER+ subtype of breast cancer on HE-BMD, and both FT and ER+ subtype of breast cancer were independent risk factors for HE-BMD. BMD is related to many factors, including alcoholism, body mass index, glucocorticoids, sex hormone levels, and thyroid and parathyroid function^43,44^. Novel mechanisms involved in the pathological process of osteoporosis have also been found, including the roles of the gut microbiome, autophagy, iron balance and cellular senescence^3^. This suggests that there are many other factors that may be associated with BMD, however, testosterone level as one of them play little mediating role in the association between breast cancer and BMD. National Health and Nutrition Examination Survey (NHANES) looked at the association between testosterone levels and BMD in middle-aged and older women from 2011 to 2016. The study, which included 2198 female participants, found a positive correlation between testosterone levels and lumbar bone density across subgroups of race and income^45^. A study of older postmenopausal women, in whom oestrogen levels were low, found that TT is directly correlated with the BMD of the LS and hip bone, and the FT level is positively correlated with the BMD of the hip^46^. Consistent with the above observational findings, our MR Study also supported the positive correlation between testosterone levels and BMD in women, and we also found a positive causal relationship between testosterone levels and the prevalence of breast cancer and its ER+ subtype. In fact, the relationship between testosterone levels and breast cancer has also been observed in prospective studies. A prospective analysis of testosterone levels and the risk of 19 types of cancer in men and women found that both FT and TT were risk factors for endometrial and breast cancer in the female population^47^. The UK Biobank included 58,629 normal-weight postmenopausal women whose increased risk of breast cancer was associated with relatively high levels of testosterone^48^. Therefore, testosterone levels have been found to have a causal relationship with both BMD and breast cancer. This relationship may explain why some observational studies have found an increase in BMD in newly diagnosed breast cancer patients^4–6^ but no causal relationship between increased BMD and breast cancer incidence. On the other hand, our MR study found that breast cancer and its ER+ subtype were potential adverse factors for BMD, while there was a positive causal relationship between testosterone level and breast cancer and its ER+ subtype. This also suggests that elevated testosterone levels may indirectly cause bone density loss while promoting breast cancer.

However, our MR Analysis also has some limitations. The polymorphism of SNPs selected as instrumental variables is a major concern. If SNPs influence multiple outcomes through independent factors, it is difficult to prove that exposure-mediated inference is unbiased. Therefore, the MR-Egger intercept and MR-PRESSO methods were used in our study to detect the level pleiotropy, in order to reduce bias. Secondly, we used the summary data of existing GWAS. Therefore, when BMD is used as the outcome, it cannot be stratified according to gender, which may lead to bias in the results. Finally, the results of this study only apply to participants of European ancestry, and further verification is needed in other populations of ancestry.

In conclusion, BMD is not a risk factor for breast cancer but breast cancer and its ER+ subtype are risk factors for BMD loss:—breast cancer might reduce HE-BMD and is considered a risk factor for osteoporosis, as is the ER+ subtype;—there is no causal relationship between BMD and breast cancer, nor is BMD mediated by FT and TT. Furthermore, higher FT and TT levels are associated with both an increased incidence of breast cancer and increased bone density:—FT and TT levels in women are risk factors for breast cancer and its ER+ subtype which have been found to be risk factors for osteoporosis (underlined above);—higher FT and TT levels increase HE-BMD and are considered protective factors for osteoporosis. So, FT and TT levels are considered to be risk factors for osteoporosis (indirectly) as well as protective factors for osteoporosis (directly).

## Supplementary Information

Supplementary Tables.

## Data Availability

GWAS summary statistics for breast cancer can be downloaded from the BCAC consortium website (http://bcac.ccge.medschl.cam.ac.uk/bcacdata/). GWAS summary statistics for BMD and hormone levels can be downloaded from three large-scale meta-analysis articles^17–19^.
