# Supplementary material for: Mendelian randomization analyses of associations between breast cancer and bone mineral density
Source: Sci Rep. 2023 Jan 31;13:1721. doi: 10.1038/s41598-023-28899-0 (PMC9889794; doi:10.1038/s41598-023-28899-0)
Supplement: Supplementary file 1 — Supplementary Tables. [file 41598_2023_28899_MOESM1_ESM.docx]

**Table S1. Results of False Discovery Rate correction.**

| Outcome/Exposure | Original P value | Adjusted P value | Significant or not |
| --- | --- | --- | --- |
| HE-BMD | | | |
| Breast Cancer | 1.01×10^-4^ | 1.51×10^-4^ | Yes |
| ER+ | 7.11×10^-5^ | 1.51×10^-4^ | Yes |
| ER- | 7.69×10^-2^ | 7.69×10^-2^ | No |
| LS-BMD | |  |  |
| Breast Cancer | 0.191 | 0.573 | No |
| ER+ | 0.410 | 0.615 | No |
| ER- | 0.706 | 0.706 | No |
| FN-BMD | |  |  |
| Breast Cancer | 0.029 | 0.087 | No |
| ER+ | 0.484 | 0.484 | No |
| ER- | 0.102 | 0.153 | No |
| FA-BMD |  |  |  |
| Breast Cancer | 0.051 | 0.153 | No |
| ER+ | 0.978 | 0.978 | No |
| ER- | 0.650 | 0.975 | No |
| Breast Cancer |  |  |  |
| HE-BMD | 0.395 | 0.549 | No |
| LS-BMD | 0.163 | 0.549 | No |
| FN-BMD | 0.412 | 0.549 | No |
| FA-BMD | 0.732 | 0.732 | No |
| ER+ |  |  |  |
| HE-BMD | 0.375 | 0.750 | No |
| LS-BMD | 0.250 | 0.750 | No |
| FN-BMD | 0.603 | 0.779 | No |
| FA-BMD | 0.779 | 0.779 | No |
| ER- |  |  |  |
| HE-BMD | 0.014 | 0.056 | No |
| LS-BMD | 0.432 | 0.581 | No |
| FN-BMD | 0.798 | 0.798 | No |
| FA-BMD | 0.436 | 0.581 | No |
| HE-BMD |  |  |  |
| FT | 2.59×10^-15^ | 1.04×10^-14^ | Yes |
| TT | 6.51×10^-5^ | 2.60×10^-4^ | Yes |
| LS-BMD |  |  |  |
| FT | 0.208 | 0.277 | No |
| TT | 0.073 | 0.102 | No |
| FN-BMD |  |  |  |
| FT | 0.759 | 0.759 | No |
| TT | 0.807 | 0.807 | No |
| FA-BMD |  |  |  |
| FT | 0.123 | 0.246 | No |
| TT | 0.076 | 0.102 | No |
| Breast Cancer |  |  |  |
| FT | 8.09×10^-4^ | 1.21×10^-3^ | Yes |
| TT | 3.87×10^-3^ | 5.81×10^-3^ | Yes |
| ER+ |  |  |  |
| FT | 8.35×10^-7^ | 2.51×10^-6^ | Yes |
| TT | 1.85×10^-4^ | 5.55×10^-4^ | Yes |
| ER- |  |  |  |
| FT | 0.896 | 0.896 | No |
| TT | 0.686 | 0.686 | No |

HE-BMD heel bone mineral density, LS-BMD lumbar spine bone mineral density, FN-BMD femoral neck bone mineral density, FA-BMD forearm bone mineral density, FT free testosterone, TT total testosterone.

**Table S2. Mendelian randomization results of IVW, weighted median, and MR-Egger from breast and its subtypes to BMD.**

|  |  | IVW | | Weighted median | | MR-Egger | |  |  | |
| --- | --- | --- | --- | --- | --- | --- | --- | --- | --- | --- |
|  | NSNP | OR (95% CI) | P | OR (95% CI) | P | OR (95% CI) | P | P_heterogeneity_ | P_pleiotropy_ | |
| HE-BMD | | | | | | | | | |  |
| Breast Cancer | 129 | 0.983(0.976,0.991) | <0.001 | \| 0.980(0.970,0.990) \| \| --- \| | <0.001 | \| 0.983(0.967,0.999) \| \| --- \| | 0.038 | <0.001 | 0.930 | |
| ER+ | 89 | 0.984(0.976,0.993) | <0.001 | \| 0.979(0.969,0.989) \| \| --- \| | <0.001 | \| 0.969(0.953,0.986) \| \| --- \| | <0.001 | <0.001 | 0.059 | |
| ER- | 21 | 0.978(0.963,0.992) | 0.003 | \| 0.981(0.967,0.996) \| \| --- \| | 0.016 | \| 1.034(0.998,1.072) \| \| --- \| | 0.077 | 0.046 | 0.004 | |
| LS-BMD | | | | |  |  |  |  |  | |
| Breast Cancer | 154 | 0.993(0.966,1.021) | 0.646 | \| 0.972(0.933,1.013) \| \| --- \| | 0.191 | \| 0.951(0.898,1.007) \| \| --- \| | 0.093 | 0.009 | 0.096 | |
| ER+ | 109 | 0.986(0.957,1.016) | 0.376 | \| 0.982(0.942,1.024) \| \| --- \| | 0.410 | \| 0.987(0.925,1.053) \| \| --- \| | 0.701 | 0.002 | 0.976 | |
| ER- | 31 | 0.992(0.952,1.033) | 0.706 | \| 1.012(0.956,1.073) \| \| --- \| | 0.664 | \| 0.995(0.880,1.126) \| \| --- \| | 0.945 | 0.343 | 0.952 | |
| FN-BMD | | | |  |  |  |  |  |  | |
| Breast Cancer | 156 | 0.984(0.961,1.007) | 0.177 | \| 0.960(0.926,0.995) \| \| --- \| | 0.029 | \| 0.960(0.915,1.007) \| \| --- \| | 0.100 | 0.040 | 0.253 | |
| ER+ | 110 | 0.994(0.969,1.020) | 0.667 | \| 0.987(0.951,1.023) \| \| --- \| | 0.484 | \| 0.978(0.926,1.034) \| \| --- \| | 0.450 | 0.003 | 0.529 | |
| ER- | 31 | 0.968(0.932,1.006) | 0.102 | \| 0.941(0.893,0.992) \| \| --- \| | 0.025 | \| 0.982(0.878,1.098) \| \| --- \| | 0.756 | 0.164 | 0.800 | |
| FA-BMD | | | |  |  |  |  |  |  | |
| Breast Cancer | 167 | 1.044(0.999,1.090) | 0.051 | \| 1.032(0.961,1.108) \| \| --- \| | 0.377 | \| 0.964(0.880,1.056) \| \| --- \| | 0.435 | 0.303 | 0.053 | |
| ER+ | 119 | 1.001(0.956,1.046) | 0.978 | \| 1.027(0.959,1.099) \| \| --- \| | 0.444 | \| 0.941(0.853,1.038) \| \| --- \| | 0.229 | 0.258 | 0.172 | |
| ER- | 34 | 0.982(0.911,1.059) | 0.650 | \| 0.974(0.883,1.074) \| \| --- \| | 0.605 | \| 0.897(0.718,1.120) \| \| --- \| | 0.345 | 0.095 | 0.398 | |

NSNP number of single nucleotide polymorphism, OR odds ratio, 95%CI lower and upper limit of 95% confidence interval, P p-value of OR, P_heterogeneity_ p-value of Cochrane’s Q value in heterogeneity test, P_pleiotropy_ p-value of MR-Egger intercept, HE-BMD heel bone mineral density, LS-BMD lumbar spine bone mineral density, FN-BMD femoral neck bone mineral density, FA-BMD forearm bone mineral density.

**Table S3. Mendelian randomization results of IVW, weighted median, and MR-Egger from BMD to breast cancer and its subtypes.**

|  |  | IVW | | Weighted median | | MR-Egger | |  |  | |
| --- | --- | --- | --- | --- | --- | --- | --- | --- | --- | --- |
|  | NSNP | OR (95% CI) | P | OR (95% CI) | P | OR (95% CI) | P | P_heterogeneity_ | P_pleiotropy_ | |
| Breast Cancer | | | | | | | | | |  |
| HE-BMD | 954 | 0.977(0.950,1.004) | 0.095 | \| 0.983(0.944,1.022) \| \| --- \| | 0.395 | \| 0.999(0.948,1.053) \| \| --- \| | 0.977 | <0.001 | 0.323 | |
| LS-BMD | 20 | 1.039(0.984,1.098) | 0.163 | \| 1.073(0.999,1.152) \| \| --- \| | 0.053 | \| 1.122(0.897,1.404) \| \| --- \| | 0.325 | 0.096 | 0.498 | |
| FN-BMD | 20 | 1.025(0.966,1.088) | 0.412 | \| 0.999(0.929,1.073) \| \| --- \| | 0.977 | \| 1.211(0.883,1.660) \| \| --- \| | 0.249 | 0.129 | 0.306 | |
| FA-BMD | 4 | 0.989(0.931,1.051) | 0.732 | 1.003(0.942,1.068) | 0.917 | 1.051(0.840,1.314) | 0.705 | 0.208 | 0.633 | |
| ER+ | | | | |  |  |  |  |  | |
| HE-BMD | 962 | 0.972(0.942,1.004) | 0.086 | \| 0.978(0.933,1.026) \| \| --- \| | 0.375 | \| 1.018(0.958,1.081) \| \| --- \| | 0.559 | <0.001 | 0.083 | |
| LS-BMD | 20 | 1.035(0.975,1.100) | 0.250 | \| 1.057(0.976,1.145) \| \| --- \| | 0.167 | \| 1.177(0.925,1.496) \| \| --- \| | 0.201 | 0.268 | 0.297 | |
| FN-BMD | 20 | 1.016(0.956,1.079) | 0.603 | \| 1.005(0.923,1.093) \| \| --- \| | 0.910 | \| 1.099(0.798,1.514) \| \| --- \| | 0.569 | 0.520 | 0.630 | |
| FA-BMD | 4 | 0.990(0.927,1.058) | 0.779 | 0.989(0.919,1.066) | 0.788 | 1.125(0.911,1.390) | 0.388 | 0.593 | 0.340 | |
| ER- | | | |  |  |  |  |  |  | |
| HE-BMD | 990 | 0.968(0.926,1.010) | 0.141 | \| 0.916(0.857,0.979) \| \| --- \| | 0.014 | \| 0.944(0.868,1.026) \| \| --- \| | 0.181 | <0.001 | 0.501 | |
| LS-BMD | 20 | 1.034(0.950,1.125) | 0.432 | \| 1.026(0.913,1.154) \| \| --- \| | 0.657 | \| 0.959(0.683,1.348) \| \| --- \| | 0.815 | 0.661 | 0.662 | |
| FN-BMD | 20 | 1.012(0.922,1.110) | 0.798 | \| 1.019(0.895,1.161) \| \| --- \| | 0.766 | \| 1.256(0.767,2.057) \| \| --- \| | 0.376 | 0.434 | 0.392 | |
| FA-BMD | 4 | 0.959(0.862,1.065) | 0.436 | 0.946(0.844,1.060) | 0.344 | 0.796(0.575,1.101) | 0.302 | 0.385 | 0.359 | |

NSNP number of single nucleotide polymorphism, OR odds ratio, 95%CI lower and upper limit of 95% confidence interval, P p-value of OR, Pheterogeneity p-value of Cochrane’s Q value in heterogeneity test, Ppleiotropy p-value of MR-Egger intercept, HE-BMD heel bone mineral density, LS-BMD lumbar spine bone mineral density, FN-BMD femoral neck bone mineral density, FA-BMD forearm bone mineral density.

**Table S4. Mendelian randomization results of IVW, weighted median, and MR-Egger from hormone levels to BMD.**

|  |  | IVW | | Weighted median | | MR-Egger | |  |  | |
| --- | --- | --- | --- | --- | --- | --- | --- | --- | --- | --- |
|  | NSNP | OR (95% CI) | P | OR (95% CI) | P | OR (95% CI) | P | P_heterogeneity_ | P_pleiotropy_ | |
| HE-BMD | | | | | | | | | |  |
| FT | 96 | 1.127(1.098,1.156) | <0.001 | \| 1.116(1.086,1.147) \| \| --- \| | <0.001 | \| 1.167(1.109,1.227) \| \| --- \| | <0.001 | <0.001 | 0.124 | |
| TT | 86 | 1.047(1.024,1.071) | <0.001 | \| 1.053(1.026,1.079) \| \| --- \| | <0.001 | \| 1.085(1.031,1.14) \| \| --- \| | 0.002 | <0.001 | 0.142 | |
| LS-BMD | | | | |  |  |  |  |  | |
| FT | 116 | 1.102(1.028,1.182) | 0.006 | \| 1.080(0.957,1.218) \| \| --- \| | 0.208 | \| 1.108(0.943,1.302) \| \| --- \| | 0.213 | 0.019 | 0.945 | |
| TT | 104 | 1.085(1.002,1.175) | 0.043 | \| 1.098(0.991,1.217) \| \| --- \| | 0.073 | \| 1.183(0.976,1.434) \| \| --- \| | 0.088 | 0.003 | 0.334 | |
| FN-BMD | | | |  |  |  |  |  |  | |
| FT | 117 | 1.055(0.991,1.123) | 0.088 | \| 1.016(0.916,1.127) \| \| --- \| | 0.759 | \| 1.157(1.004,1.334) \| \| --- \| | 0.045 | 0.006 | 0.160 | |
| TT | 103 | 1.049(0.977,1.126) | 0.181 | \| 1.011(0.922,1.109) \| \| --- \| | 0.807 | \| 1.218(1.027,1.444) \| \| --- \| | 0.025 | <0.001 | 0.062 | |
| FA-BMD | | | |  |  |  |  |  |  | |
| FT | 122 | 1.254(1.106,1.423) | <0.001 | \| 1.182(0.955,1.464) \| \| --- \| | 0.123 | \| 1.197(0.894,1.603) \| \| --- \| | 0.229 | 0.001 | 0.726 | |
| TT | 112 | 1.196(1.046,1.368) | 0.009 | \| 1.174(0.982,1.403) \| \| --- \| | 0.076 | \| 1.035(0.748,1.434) \| \| --- \| | 0.833 | 0.004 | 0.342 | |

NSNP number of single nucleotide polymorphism, OR odds ratio, 95%CI lower and upper limit of 95% confidence interval, P p-value of OR, Pheterogeneity p-value of Cochrane’s Q value in heterogeneity test, Ppleiotropy p-value of MR-Egger intercept, HE-BMD heel bone mineral density, LS-BMD lumbar spine bone mineral density, FN-BMD femoral neck bone mineral density, FA-BMD forearm bone mineral density, FT free testosterone, TT total testosterone.

**Table S5. Mendelian randomization results of IVW, weighted median, and MR-Egger from hormone levels to breast cancer and its subtypes.**

|  |  | IVW | | Weighted median | | MR-Egger | |  |  | |
| --- | --- | --- | --- | --- | --- | --- | --- | --- | --- | --- |
|  | NSNP | OR (95% CI) | P | OR (95% CI) | P | OR (95% CI) | P | P_heterogeneity_ | P_pleiotropy_ | |
| Breast Cancer | | | | | | | | | |  |
| FT | 130 | 1.144(1.084,1.208) | <0.001 | \| 1.137(1.054,1.226) \| \| --- \| | <0.001 | \| 1.191(1.062,1.336) \| \| --- \| | 0.003 | <0.001 | 0.435 | |
| TT | 117 | 1.134(1.075,1.196) | <0.001 | \| 1.130(1.040,1.227) \| \| --- \| | 0.004 | \| 1.227(1.108,1.359) \| \| --- \| | <0.001 | <0.001 | 0.081 | |
| ER+ | | | | |  |  |  |  |  | |
| FT | 132 | 1.198(1.124,1.278) | <0.001 | \| 1.235(1.136,1.344) \| \| --- \| | <0.001 | \| 1.273(1.113,1.456) \| \| --- \| | <0.001 | <0.001 | 0.321 | |
| TT | 119 | 1.188(1.112,1.268) | <0.001 | \| 1.213(1.096,1.343) \| \| --- \| | <0.001 | \| 1.319(1.163,1.496) \| \| --- \| | <0.001 | <0.001 | 0.058 | |
| ER- | | | |  |  |  |  |  |  | |
| FT | 130 | 0.979(0.898,1.067) | 0.629 | \| 1.009(0.881,1.154) \| \| --- \| | 0.896 | \| 1.028(0.857,1.234) \| \| --- \| | 0.760 | 0.002 | 0.544 | |
| TT | 124 | 0.985(0.906,1.071) | 0.736 | \| 0.971(0.844,1.117) \| \| --- \| | 0.686 | \| 0.994(0.845,1.170) \| \| --- \| | 0.950 | 0.033 | 0.897 | |

NSNP number of single nucleotide polymorphism, OR odds ratio, 95%CI lower and upper limit of 95% confidence interval, P p-value of OR, Pheterogeneity p-value of Cochrane’s Q value in heterogeneity test, Ppleiotropy p-value of MR-Egger intercept, FT free testosterone, TT total testosterone.
